# Lifetime risk of cancer in carriers of intermediate alleles in the *HTT* gene

**DOI:** 10.1038/s41598-026-35941-4

**Published:** 2026-01-16

**Authors:** Jimmy Sundblom, Ingvar Bergdahl, Eva-Lena Stattin, Valter Niemelä

**Affiliations:** 1 Department of Medical Sciences, Neurosurgery, Uppsala University, Uppsala, Sweden; 2 Section of Sustainable Health, Department of Public Health and Clinical Medicine, Umeå University, Umeå, Sweden; 3 Department of Immunology Genetics and Pathology; Genetics, Uppsala University, Uppsala, Sweden; 4 Department of Medical Sciences, Neurology, Uppsala University, Akademiska sjukhuset, ing 85, Uppsala, 751 85 Sweden

**Keywords:** Huntington´s disease, Cancer, Intermediate alleles, Reduced penetrance alleles, Genetics, Cancer epidemiology, Cancer genetics, Epigenetics, Medical genetics, Neuroscience

## Abstract

Previous studies have found a markedly reduced risk of cancer among Huntington’s disease (HD) patients with CAG ≥ 40, but data on cancer risk at shorter repeat numbers are lacking. The study includes 8149 subjects from Northern Sweden Health and Disease Study. Genotyping yielded a large number of intermediate allele carriers (IA, CAG_n_ 27–35, (*n* = 497), normal alleles (CAG_n_ 17–26,*n* = 6584), short alleles (CAG ≤ 16, *n* = 169) and 31 subjects with > 35 repeats, including reduced penetrance alleles (36–39; not guaranteed to suffer HD symptoms during a normal lifespan) and HD alleles > 39. Cancer diagnoses were retrieved from the Swedish Cancer Registry and the Hospital Discharge Registry and death certificates. We used Kaplan-Meier curves and Cox proportional hazard models to estimate the time to cancer, on strata of the population created by CAG repeat number intervals. Smoking status, BMI, as well as alcohol consumption were included in the models. 2735 participants (33.6%) had ≥ 1 cancer type. The Hazard-Ratio (HR) for IA carriers compared with normal alleles was similar, 0.97 CI 0.82–1.15). The reduced penetrance allele group (CAG_n_ 36–39, *n* = 29) had HR of 0.54 CI 0.22–1.30 similar to what has been reported with a full penetrance allele. Intermediate allele carriers as a group did not have a reduced risk of cancer. It remains possible that reduced penetrance alleles confer lower risk of cancer, with signs of a dose-dependent protective effect of CAG repeat length. The latter finding needs to be confirmed in even larger cohorts as these repeat numbers are relatively rare.

## Introduction

Huntington’s disease (HD) is a hereditary neurodegenerative disorder caused by the expansion of CAG trinucleotide repeats within the huntingtin gene (HTT). While the presence of a full mutation (≥ 40 CAG repeats) is well-established as the cause of HD, the health significance of carrying intermediate alleles (IA) —CAG repeat lengths ranging from 27 to 35, remains less studied. IA: s were initially considered non-pathogenic, but emerging evidence suggests their potential impact on disease manifestations similar to that of HD in rare cases^1^. Increased lifetime risk of depression has also been reported for individuals with short or long normal alleles^2^.

Many different trinucleotide disorders have been described, and repeated sequences are prevalent in the human genome^9^. They give rise to disease in different ways, including loss of function, toxic RNA gain of function and repeat sequences translated into pathogenic proteins (as in HD)^10^.

Research on the effects of genomic repeat sequences may reveal new sources of human phenotypic variability.

Interestingly, several studies have rendered evidence supporting an association between HD and decreased risk of cancer^3–6^. The studies that had access to genotype found no association between CAG repeat number and cancer occurrence^5,6^. Only one study has reported a modulating effect of IAs (CAG_n_ 27–35) on the risk of developing cancer. This study focused on a cohort of BRCA mutation carriers and found a decreased risk of breast cancer among BRCA carriers who also have an IA^7^. Prospective studies on IA carriers and their risk of all cancers are lacking. Also, the risk among persons carrying reduced penetrance alleles (not guaranteed to cause symptoms of HD during a normal lifespan) with 36–39 CAG repeats is not known.

The SHAPE study (Swedish-Huntingtin-Alleles-PhenotypE) was designed to investigate the prevalence and potential health consequences of IAs in the general population in Northern Sweden.

As we have previously reported, the prevalence of IAs in this region is 6.8%^8^. With this high occurrence of IAs, it is important to understand their role in disease susceptibility.

Many different trinucleotide disorders have been described, and repeated sequences are prevalent in the human genome^9^. They give rise to disease in different ways, including loss of function, toxic RNA gain of function and repeat sequences translated into pathogenic proteins (as in HD)^10^.

Research on the effects of genomic repeat sequences may reveal new sources of human phenotypic variability.

Here, we will explore the potential association between IAs and risk of cancer. The primary aim of this study is to investigate if carrying an IA confers a reduced lifetime risk of cancer. Secondary aims include assessing association of CAG repeat length with the risk of all cancers, specific cancer types/locations as well as risk of repeated cancer.

## Materials and methods

### Study population and baseline data

Details concerning the study cohort and design have previously been published^8^. Briefly, the SHAPE study utilizes a population sample from the Northern Sweden Health and Disease Study (NSHDS) in the counties of Norrbotten and Västerbotten. The blood samples from a nested-case control study examining first myocardial infarctions and demographically matched controls (ratio 1:2) were-used in this study.

DNA was genotyped using PCR based analysis and fragment sizing at SciLife Lab at Uppsala University to measure the CAG repeat number of both alleles in the *HTT-*gene. Samples that could not be genotyped due to technical difficulties, were categorized as a separate group in the analysis to verify that the error was random and unrelated to CAG repeat number.

Exclusion of participants: Statistics Sweden flagged social security numbers that were re-used and participants that have had multiple social security numbers, which may cause difficulty in correctly linking the number to an individual throughout the observation period of the study. Further, exclusion of participants that either migrated to Sweden after the age of 30, and those who spent > 10 years abroad was deemed necessary. This was done in order to exclude participants that may have had cancer abroad that would not have been recorded in the Swedish Cancer register.

### Extraction of patient data from National registries in Sweden

The study population was linked to several national registries by Statistics Sweden using individual national identification numbers that were replaced by serial numbers in the study data to prevent identification. All cancer diagnoses were extracted from The Cancer Registry until the 31st of December 2020. The Swedish Cancer Register was founded in 1958 and covers the whole population. All benign and malignant tumors diagnosed in the country are included by clinicians and pathologists. A limitation in the Swedish Cancer Registry is that some older patients who had cancer diagnosed near death may have been underreported. To mitigate this, cancer cases were identified in The Death Certificates Registry (1985–2020), but only those also verified in the Hospital Discharge Registry or Outpatient Registry were included. If cancer was only indicated by the death certificate this was not deemed sufficient to confirm a malignant disease and therefore not included as a cancer case. The Hospital discharge registry has complete coverage in the regions of Västerbotten and Norrbotten since 1984 and from this year on cancer diagnoses were included in the present study. The Outpatient Registry was launched in 1997 and therefore malignant diagnoses between 1997 and 2020 were included. Both registries were searched using the International Classification of Diseases (ICD) diagnostic codes for malignancies as well as neoplasms of uncertain or unknown behavior as follows; 140–209 and 210–228 in ICD-8 (year 1969–1986); 140–208 and 230–239, for ICD-9 (year 1987–1996); and C00-D09 and D37-D48 in ICD-10 (1997-).

Individual level data was also linked from The Emigration Registry (for date of migration and emigration) and the Swedish National Population and Housing Census (for educational level).

### Statistical analysis

The dataset was divided into sub-groups using CAG repeat number intervals (CAG_n_ of the longest allele). We used Kaplan-Meier curves to illustrate- and Cox regression models to estimate the risk of cancer diagnosis over time compared between CAG interval sub-groups. Kaplan-Meier curves and Cox proportional hazard models were created for the following known risk factors for cancer; smoking status^11,12^, alcohol consumption^13^, BMI^14^, and sex^15^. All of these risk factors were adjusted for in the final Cox regression model.

Asymptotic linear-by-linear association test was used to test the null hypothesis of independence between CAG repeat intervals and cancer incidence.

## Results

From the original cohort^8^ totally 121 participants were removed, 25 due to issues such as uncertain ID, misdiagnosis of myocardial infarction, or withdrawal of consent. Twenty-eight participants that may have had more than one social security number were flagged by Statistics Sweden and were excluded from the study. Five participants that had re-used social security numbers were also excluded. Participants that migrated to Sweden after the age of 30 were excluded (*n* = 46) as well as those who had stayed a period of > 10 years abroad (*n* = 17).

After exclusion of the participants above the study population (n = 8149) was stratified according to CAG repeat number (CAG_n_) as follows; “short alleles” (CAG_n_ ≤16, n = 169), ”normal alleles” (CAG_n_ 17–26, *n* = 6584), “intermediate alleles” (CAG_n_ 27–35, *n* = 497), “reduced penetrance alleles” (CAG_n_ 36–39, *n* = 29), and “full penetrance alleles” (CAG_n_ ≥40 repeats, *n* = 2). A final category “missing genotype”, consisted of 868 participants.

Roughly half of the study population (50,4%) was alive at the end of the observation period (31st of December 2020), with a mean age of 77.4 years (SD 8.53). 67% (67.5%) were males. Median body mass index (BMI) at enrollment in the study was 26.0 kg/m2. Current or previous cigarette smoking habit was common (52%). The most common educational status was upper secondary level (45.6%). See Table 1.

### Prevalence of cancer in the population

The linkage with the national registries detected 2735 participants (33.6%) with (≥ 1) cancer diagnosis during the follow-up period. Additionally, 71 participants who were not retrieved in the Swedish Cancer Registry had a cancer diagnosis on their death certificate of which 55 cases could be verified in the Hospital Discharge Registry and were subsequently included in the analysis.

The short repeat group (CAG < 17), normal allele group (CAG 17–26) as well as IA group (CAG 27–35) all had similar proportions affected by cancer during the observation period (33.7%, 33.7%, and 33.0% respectively), while the relatively small reduced penetrance allele group (CAG 36–39, *n* = 29) had fewer cancer diagnoses (17.2%). Patient characteristics and data summarized in Table 1.

**Table 1:** Characteristics of the study population.

		Overall	≤ 16	17–26	27–35	36–39	≥ 40	Missing
n		8149	169	6584	497	29	2	868
Long allele (median [IQR])		19.0 [17.0, 22.0]	16.0 [15.0, 16.0]	19.0 [17.0, 21.0]	28.0 [27.0, 29.0]	37.0 [36.0, 37.0]	41.0 [40.5, 41.5]	NA [NA, NA]
Gender (%)	Female	2645 (32.5)	52 (30.8)	2173 (33.0)	168 (33.8)	6 (20.7)	1 (50.0)	245 (28.2)
	Male	5504 (67.5)	117 (69.2)	4411 (67.0)	329 (66.2)	23 (79.3)	1 (50.0)	623 (71.8)
Born year (%)	1920–1932	2034 (25.0)	48 (28.4)	1622 (24.6)	112 (22.5)	6 (20.7)	0 (0.0)	246 (28.3)
	1933–1938	1930 (23.7)	47 (27.8)	1543 (23.4)	118 (23.7)	7 (24.1)	0 (0.0)	215 (24.8)
	1938–1943	2020 (24.8)	31 (18.3)	1657 (25.2)	134 (27.0)	8 (27.6)	2 (100.0)	188 (21.7)
	1944–1967	2165 (26.6)	43 (25.4)	1762 (26.8)	133 (26.8)	8 (27.6)	0 (0.0)	219 (25.2)
BMI, median [IQR])		26.0 [23.9, 28.5]	26.3 [23.9, 28.7]	26.0 [23.9, 28.4]	26.0 [23.7, 28.4]	26.7 [24.1, 29.9]	25.3 [22.9, 27.7]	26.0 [23.9, 28.5]
Education level ISCED 2011 (%)	Higher (ISCED ≥ 4	883 (21.6)	21 (25.3)	741 (22.3)	52 (20.9)	3 (21.4)	0 (0.0)	66 (16.2)
	Upper secondary (ISCED 3)	1861 (45.6)	34 (41.0)	1522 (45.8)	112 (45.0)	6 (42.9)	2 (100.0)	185 (45.3)
	Lower secondary / ISCED 2	1335 (32.7)	28 (33.7)	1060 (31.9)	85 (34.1)	5 (35.7)	0 (0.0)	157 (38.5)
	NA							
Alcohol consumption CAGE (%)	No use	993 (12.2)	20 (11.8)	778 (11.8)	69 (13.9)	3 (10.3)	0 (0.0)	123 (14.2)
	No risk 0–1 p	3886 (47.7)	82 (48.5)	3177 (48.3)	234 (47.1)	14 (48.3)	1 (50.0)	378 (43.5)
	Risk use 2–4 p	424 (5.2)	5 (3.0)	352 (5.3)	19 (3.8)	3 (10.3)	1 (50.0)	44 (5.1)
	Form not provided	2436 (29.9)	56 (33.1)	1969 (29.9)	148 (29.8)	8 (27.6)	0 (0.0)	255 (29.4)
	Inconsequent/ no answer	410 (5.0)	6 (3.6)	308 (4.7)	27 (5.4)	1 (3.4)	0 (0.0)	68 (7.8)
Smoking status (%)	No use	2714 (33.3)	51 (30.2)	2150 (32.7)	178 (35.8)	7 (24.1)	1	327 (37.7)
							(50.0)	
	Previous use	2428 (29.8)	59 (34.9)	1947 (29.6)	144 (29.0)	9 (31.0)	0 (0.0)	269 (31.0)
	Current use	1811 (22.2)	36 (21.3)	1484 (22.5)	103 (20.7)	8 (27.6)	1 (50.0)	179 (20.6)
	Form not provided	942 (11.6)	19 (11.2)	800 (12.2)	55 (11.1)	4 (13.8)	0 (0.0)	64 (7.4)
	Inconsequent/ no answer	254 (3.1)	4 (2.4)	203 (3.1)	17 (3.4)	1 (3.4)	0 (0.0)	29 (3.3)
Cancer (%)	Yes	2735 (33.6)	57 (33.7)	2219 (33.7)	164 (33.0)	5 (17.2)	0 (0.0)	290 (33.4)
Multiple organ/ type (%)	More than 1	435 (15.9)	11 (19.3)	344 (15.5)	36 (22.0)	1 (20.0)	0 (NaN)	43 (14.8)
	Only 1	2301 (84.1)	46 (80.7)	1876 (84.5)	128 (78.0)	4 (80.0)	0 (NaN)	247 (85.2)
Cancer, age at diagnosis (median [IQR])		70.5 [63.4, 76.6]	71.8 [66.1, 77.2]	70.3 [63.4, 76.3]	69.8 [63.2, 77.2]	70.9 [67.8, 77.5]	NA [NA, NA]	72.3 [64.1, 77.6]
Death (%)	Yes	4057 (49.8)	86 (50.9)	3249 (49.3)	247 (49.7)	15 (51.7)	0 (0.0)	460 (53.0)
	No	4092 (50.2)	83 (49.1)	3335 (50.7)	250 (50.3)	14 (48.3)	2 (100.0)	408 (47.0)
Years first cancer to death (median [IQR])		4.11 [0.9, 11.2]	5.13 [1.9, 15.8]	4.13 [0.9, 11.1]	3.85 [1.0, 12.3]	3.67 [2.4, 5.1]	NA [NA, NA]	3.71 [0.9, 11.1]
Age at death (median [IQR])		77.7 [70.6, 83.7]	77.4 [72.9, 84.5]	77.6 [70.4, 83.6]	79.2 [72.5, 84.7]	75.2 [68.5, 83.1]	NA [NA, NA]	77.8 [71.2, 83.5]

Smoking habits, alcohol consumption and body mass index (BMI) were recorded at inclusion in the northern Swedish study of health and disease (NSHDS) whereas the remaining data was extracted from national registries in Sweden. CAG repeat length was analyzed from blood samples donated at inclusion in the study.

### Types of cancer

Prostate cancer (*n* = 725) was the most common entity, followed by colorectal (*n* = 339), gastrointestinal (*n* = 212), urinary tract/renal (*n* = 191), hematological malignancies (*n* = 179) and breast cancer (*n* = 155), see Table 2 for summary. The panorama is in accordance with published data regarding cancer incidence in Sweden^16^.

**Table 2:** Cancer distribution in the SHAPE-cohort stratified by CAG repeat number.

		Overall	≤ 16	17–26	27–35	36–39	≥ 40	Missing
n		8149	169	6584	497	29	2	868
Cancer (%)	Yes	2735 (33.6)	57 (33.7)	2219 (33.7)	164 (33.0)	5 (17.2)	0 (0.0)	290 (33.4)
Cancer (first type) (%)	Blood	179 (6.5)	5 (8.8)	139 (6.3)	16 (9.8)	0 (0.0)	0 (NaN)	19 (6.6)
	Breast	155 (5.7)	2 (3.5)	135 (6.1)	8 (4.9)	0 (0.0)	0 (NaN)	10 (3.4)
	Colorectal	339 (12.4)	11 (19.3)	276 (12.4)	20 (12.2)	1 (20.0)	0 (NaN)	31 (10.7)
	Gastrointestinal	212 (7.7)	1 (1.8)	181 (8.2)	8 (4.9)	0 (0.0)	0 (NaN)	22 (7.6)
	Female organs	213 (7.8)	5 (8.8)	166 (7.5)	16 (9.8)	0 (0.0)	0 (NaN)	26 (9.0)
	Lung	130 (4.8)	0 (0.0)	104 (4.7)	11 (6.7)	0 (0.0)	0 (NaN)	15 (5.2)
	Other	592 (21.6)	10 (17.5)	483 (21.8)	38 (23.2)	0 (0.0)	0 (NaN)	61 (21.0)
	Prostate	725 (26.5)	20 (35.1)	581 (26.2)	41 (25.0)	0 (0.0)	0 (NaN)	83 (28.6)
	Urinary tract or Renal	191 (7.0)	3 (5.3)	155 (7.0)	6 (3.7)	4 (80.0)	0 (NaN)	23 (7.9)
Cancer, age at diagnosis (median [IQR])		70.5 [63.4, 76.6]	71.8 [66.1, 77.2]	70.3 [63.4, 76.3]	69.8 [63.2, 77.2]	70.9 [67.8, 77.5]	NA [NA, NA]	72.3 [64.1, 77.6]
Long allele (median [IQR])		19.0 [17.0, 22.0]	16.0 [15.0, 16.0]	19.0 [17.0, 21.0]	28.0 [27.0, 29.0]	37.0 [36.0, 37.0]	41.0 [40.5, 41.5]	NA [NA, NA]

### Influence of CAG repeat number on time to first cancer

**Fig. 1:**
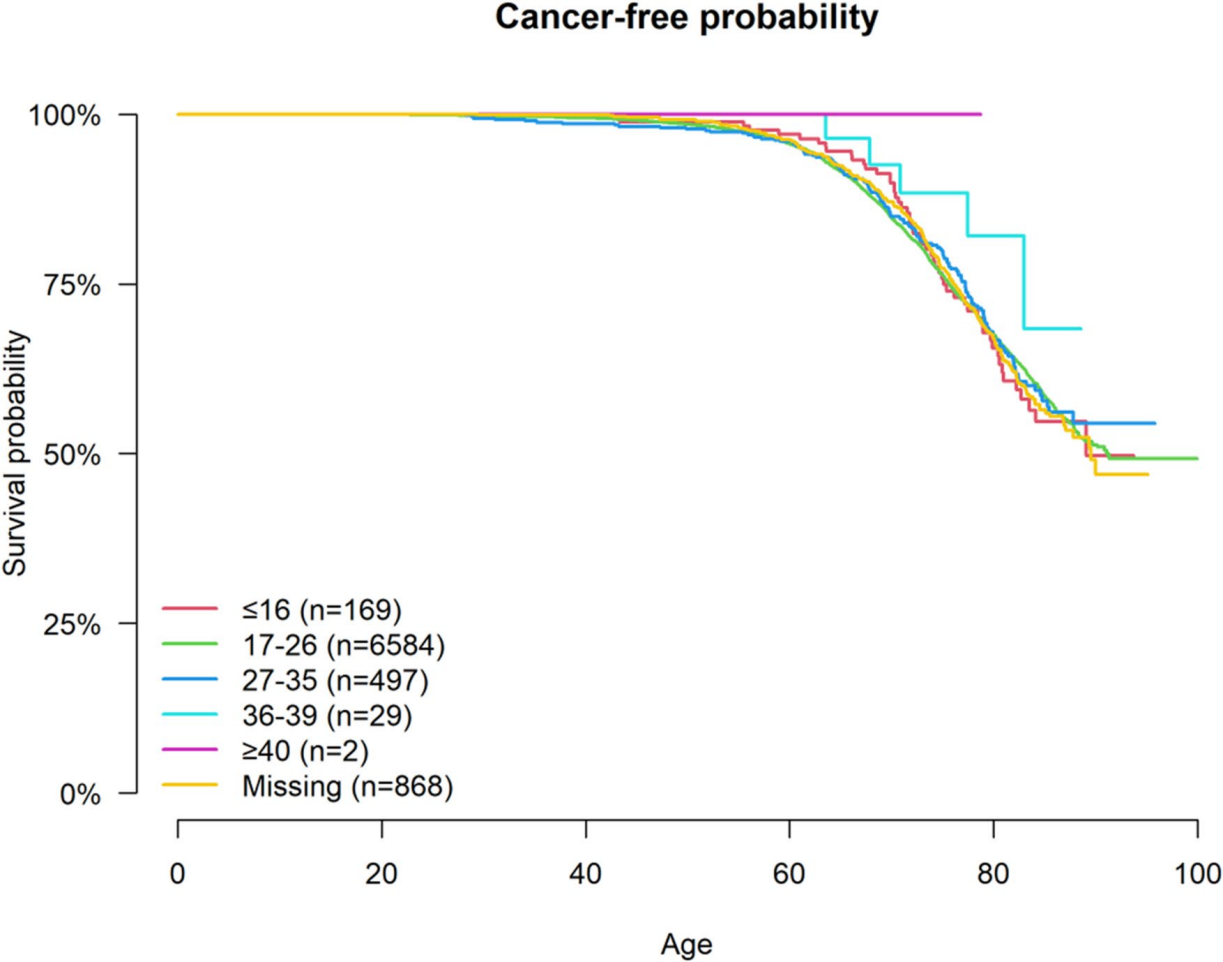
The Kaplan-Meier plot displays the time from birth to first cancer diagnosis for the respective CAG repeat intervals. The Cox regression model identified no significant differences between the groups, although the reduced penetrance allele group (36–39 CAG) shows a trend to longer survival without cancer.

Time from birth to first cancer diagnosis was not significantly different compared between the CAG repeat groups. Before adjusting for any risk factors the hazard-ratio (HR) for IA carriers compared to normal alleles was similar (HR = 0.96 CI 0.81–1.14). While the reduced penetrance allele group had a lower HR of 0.56 (CI 0.23–1.34), this was not statistically significant. Cancer-free probability at age presented group-wise in Fig. 1.

Further we assessed known risk factors for cancer using unadjusted Cox proportional hazard models. This confirmed a statistically significant relationship between current- and previous smoking and increased cancer risk (*p* < 0.001). Abstaining from alcohol was associated with reduced risk of cancer compared to normal consumption, CAGE index = 0–1 (*p* = 0.004). The completeness of questionnaire data varied due to differing practices across the three original cohorts that were included in the study population. First, the participants from the largest cohort VIP (*n* = 6405) were all inquired about smoking, and only a small proportion were not provided the alcohol questionnaire. Notably, the participants who were not provided both the questionnaires for alcohol and smoking, were exclusively women from MSP (*n* = 942), showing a tendency to lower cancer risk compared to those with complete data (*p* = 0.052). Conversely, the participants who were not provided only the alcohol questionnaire included predominantly men with a higher representation from MONICA compared to those with complete data. This group also displayed a significantly lower risk of cancer (*p* < 0.001). Overweight and obesity were associated with increased cancer risk compared to normal weight (respectively *p* = 0.003 and *p* = 0.002). Kaplan-Meier curves indicated higher cancer risk for women than men early in life, whereas males had higher risk than women later in life, meaning that gender did not fulfill criteria for proportional hazards.

Consequently, we stratified the model for sex and included alcohol consumption, smoking status, and BMI. This hardly changed the risk estimates for the CAG repeat groups compared to the risk with normal alleles, HR after adjustment was 0.97 for the IA group and 0.54 for the reduced penetrance allele group. There was also no difference in risk between the missing allele group and the normal allele group. Hazard-ratios are presented in Table 3.

**Table 3:** Cox regression: time to cancer based on CAG-repeat length.

Characteristic	*N*	Cases	HR^1^	95% CI^1^	*p*-value
Participants	8147	2477			
17–26			—	—	
< 17			0.98	0.75, 1.29	0.9
27–35			0.97	0.82, 1.15	0.7
36–39			0.54	0.22, 1.30	0.2
40+					
Missing			1.01	0.89, 1.15	0.9

Variable count by gender group			
	Overall	Female	Male
n	8147	2644	5503
CAG interval (%)			
17–26	6584 (80.8)	2173 (82.2)	4411 (80.2)
≤ 16	169 (2.1)	52 (2.0)	117 (2.1)
27–35	497 (6.1)	168 (6.4)	329 6.0)
36–39	29 (0.4)	6 (0.2)	23 (0.4)
Missing	868 (10.7)	245 (9.3)	623 (11.3)

Model stratified for sex and including the following risk factors; alcohol consumtion, smoking habits and BMI.

^1^HR = Hazard Ratio, CI = Confidence Interval

A Linear-by-linear test indicated no significant association between the respective CAG repeat intervals and cumulative types or organs affected by cancer (*p* = 0.374, Table 4). Similarly there was no correlation between the two variables (Spearman *R* = -0.0085).

**Table 4:** CAG repeat intervals vs. cumulative cancer types/locations.

	≤ 16	17–26	27–35	36–39	≥ 40
n =	169	6584	497	29	2
Cumulative cancer types/locations (%)					
0	116 (68.6)	4579 (69.5)	349 (70.2)	24 (82.8)	2 (100.0)
1	46 (27.2)	1742 (26.5)	127 (25.6)	4 (13.8)	0 (0.0)
2	6 (3.6)	242 (3.7)	19 (3.8)	1 (3.4)	0 (0.0)
3	1 (0.6)	21 (0.3)	2 (0.4)	0 (0.0)	0 (0.0)

There was no significant association between CAG repeat number and cancer risk (all cancers) (HR 1.006 95% CI 0.990–1.023, *p* = 0.437).

For cancers with significant occurrences, chi^2−^test was done to assess significant (*p* = 0.05) differences between the normal alleles and the IA groups. Statistically significant risk reduction was found in urinary tract cancers (*p* = 0.0047) and gastrointestinal cancers (*p* = 0.0088).

**Fig. 2:**
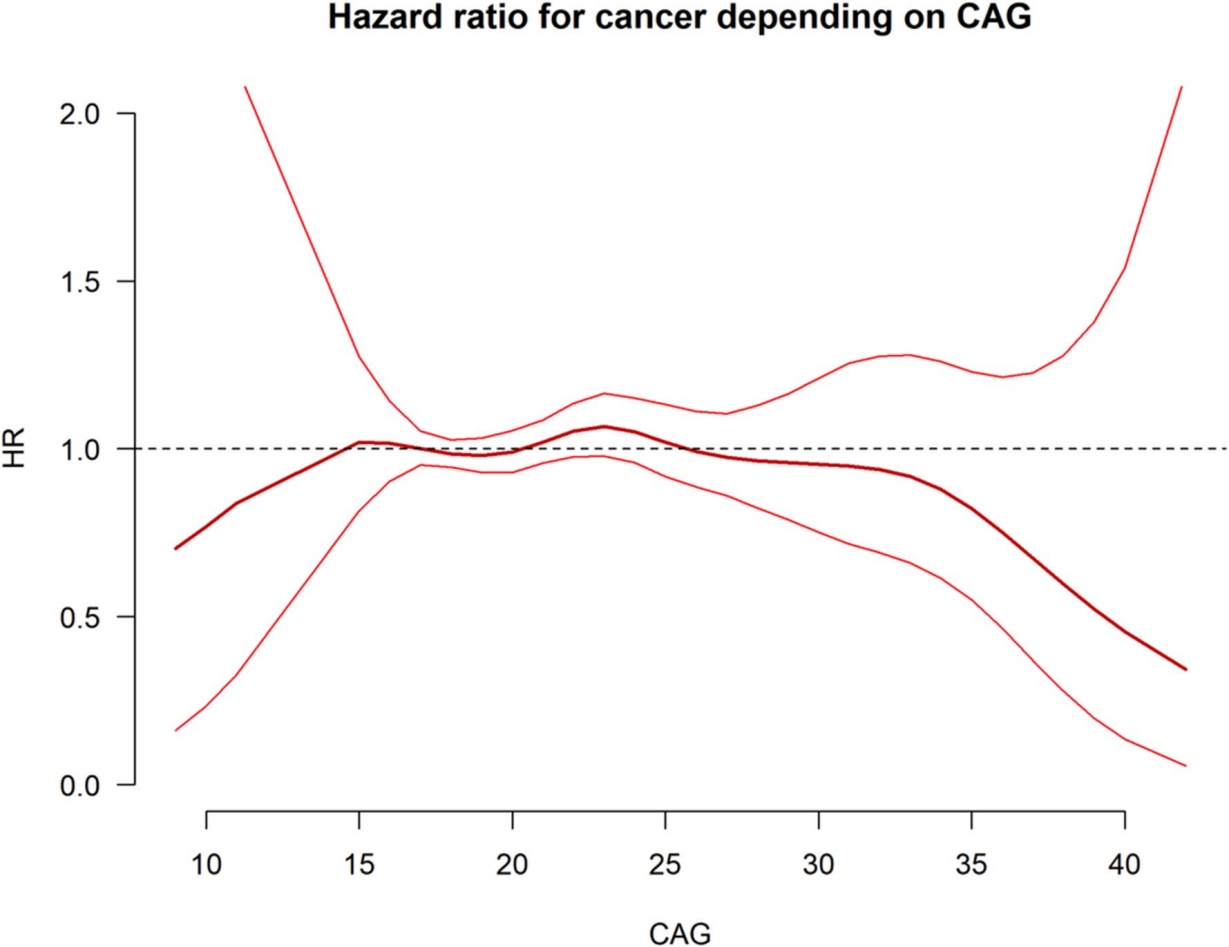
Cox regression with psplines to examine the hazard ratio for cancer in relation to CAG repeat number. This model can be used to visually identify a threshold value. Both the central bold line and the (thin red) psplines should intersect the HR = 1 line, in order to determine a threshold, however this did not occur. Nevertheless, the mean HR (represented by the bold red line) indicates a threshold around 30–35 CAG repeats as well as a cubic relationship between CAG number and cancer risk, though this was not statistically significant. A divergence of psplines is observed where the number of individuals with corresponding CAG numbers decrease.

A previous study found an association between the cubic function of CAG and lifetime risk of depression^2^. As our present findings in Fig. 2 similarly visually suggest such a relationship we statistically tested this hypothesis for cancer risk and the quadratic term for CAG. The model could not confirm a reduced risk of cancer for short and long alleles, (HR 0.998 95% CI 0.996–1.001, *p* = 0.160).

## Discussion

Several studies have found dramatically reduced risk of cancer among HD patients^3,4,6^, but there is a lack of knowledge concerning slightly prolonged HTT alleles, in particular the prevalent IAs. This is the first study of the overall risk of cancer in carriers of IAs. The results imply that there is no difference in risk of cancer between carriers of intermediate versus normal alleles (HR = 0.97 CI 0.82–1.15). This is relevant information as IAs are common in many populations (for instance 6.8% in the present cohort). Although the study was not powered to assess cancer risk among the reduced penetrance allele group, the HR of 0.54 (compared to normal alleles) is strikingly similar to what has been reported for full penetrance alleles (CAG ≥ 40).

Previous studies have almost exclusively included Huntington’s disease patients with full penetrance alleles (CAG ≥ 40). None of these have identified a threshold CAG number for risk reduction or any influence of the CAG repeat number on cancer risk.

Our new findings combined with the known risk reduction for cancer from previous research on full penetrance alleles suggests that a threshold for the CAG length lies above or in the upper range of IAs. However, our statistical analysis could not identify such a threshold, likely due to the limited number of subjects included with larger CAG repeat numbers. Similarly, we could not confirm direct correlation between cancer risk and CAG repeat number. Another possibility is that the cancer risk is best explained by a quadratic function of CAG length as has been showed regarding the risk for depression^2^ as well as intelligence^17^. This model, although not statistically significant, showed better performance compared with direct CAG number in our study population.

Although previous studies have assessed the risk of cancer, none of these have assessed whether there are also fewer repeated cancer cases among *HTT*-gene expansion carriers. In this study we found no evidence to support that IA carriers would have lower risk of cancer in multiple locations or different types.

A remaining question is whether varying CAG length modulates various and disparate human biological functions (such as affective disorders, cognition, cancer susceptibility) through the same dose-response mechanisms.

Regarding differential risk modulation for specific cancer types mediated by HTT CAG expansions the previous studies have yielded no clear consensus. For instance, Coarelli et al. found reduced prevalence of all cancers except skin cancers (melanoma) in French HD patients^5^. Sorensen and colleagues in Denmark reported overall reduced risk of cancers except for the oral- and pharyngeal types^3^. Ji et al. examined a population of 1510 Swedish HD patients, and found significantly lower risk for all cancers, except colorectal and urinary system^4^. McNulty et al. analyzed the European Registry study population finding 173 cancers in 6528 HD patients with SIR 0.26 (CI 0.22–0.3) for all cancers, and the lowest HR were found in colorectal and prostate cancer^6^. In contrast a study from the global Enroll-HD cohort with a relatively short observation period did not confirm a reduced risk of cancer in 2608 HD patients. The cancer subtypes were not analyzed in the latter study^18^. Consequently, there is largely an agreement on the overall risk reduction for cancer but not whether the extended polyglutamine tract specifically inhibits growth of some cancers more than others. The findings of a reduced risk for urinary tract malignancies and gastrointestinal cancers in this study must be interpreted with caution due to risk of multiple testing, but it may warrant further investigation. Of note, the distribution of cancer types/locations in the present study population matched the overall pattern of malignancies in Sweden^16^.

The reduced cancer incidence associated with an expanded trinucleotide sequence of the *HTT* gene may be due to shared molecular pathways between cancer and HD. The CAG expansion in HD causes increased levels of p53 which leads to degradation and decreased levels of HSF1 protein, contrary to findings of decreased p53 and increased HSF1 in most tumor cells^19^. Small interfering RNAs derived from the CAG repeat sequence in HD are highly toxic to cancer cells and have been suggested as potential therapies for cancer^20^.

### Limitations

The study was well powered to assess differences in overall risk of cancer between carriers of IAs and normal alleles but not large repeat sizes (CAG ≥ 36) or very small repeats. The results should be considered bearing this in mind.

The study population, in contrast to previous studies on the topic, includes no, or very few, individuals with HD. This limits bias by underreporting cancer due to the disease. The reason we believe that no, or very few, HD cases were included is that NSHDS enrolled middle-aged individuals from the general population.

The individuals included in this study were from a case-control study of myocardial infarction (MI) nested within NSHDS, but we do not believe this has biased the study results. One third later in life became cases of first myocardial infarction (MI) and the others were matched controls but, importantly, there was no difference in the distribution of IAs between MI cases and controls^8^. Therefore we believe this population in regard to the distribution of CAG repeats reflects the background population.

Although failure to genotype was likely caused by random errors, individuals with such missing data were included as a separate group in the study. As the cancer risk as well as other participant data in the missing allele group was not different from normal alleles, this suggests the failure of genotyping was not related to CAG-repeat length outliers.

Due to lack of information on cancer therapies no analysis of treatment response or prognosis could be conducted.

As the Swedish Cancer Registry only includes cancer cases diagnosed in Sweden there is a risk that cancers diagnosed abroad may not be recorded in the study. We minimized this risk by excluding the few participants who spent more than ten years abroad or who immigrated to Sweden after the age of 30. Individuals who moved abroad without returning could still be included but were censored at the date of migration.

Whereas previous studies in this field that utilized HD cohorts have a risk of underestimating cancer cases due to the serious neurological disorder, we believe there was no such risk concerning the present study population as it was not selected for HD subjects. Further, as the Swedish cancer registry was founded in 1958, well after the oldest participants in the SHAPE study reached adulthood, some historic cancer cases with early onset may not have been recorded. This however, does not affect the comparison of cancer risk depending on CAG repeat number and the cancer incidence in this population was representative of published data on cancer occurrence in Sweden^16^.

The SHAPE study cohort was created using the NSHDS that combines participants from three sub-cohorts with slightly differing protocols.

Cases with missing data on risk factors for cancer were still included in the multiple regression model not to exclude a significant part of the population.

## Conclusion

There was no reduced risk of cancer among the IA carriers, but we cannot exclude that reduced penetrance alleles confer a reduced risk of cancer. In order to identify where the threshold CAG repeat number for reduced cancer risk lies, it might be suitable to study a very large cohort selected for CAG repeat numbers around 30.

## Data Availability

The data underlying this article cannot be shared publicly due to the genetic and comprehensive demographic nature of it. The data will be shared on reasonable request to the corresponding author.
